# The Assembly of Flagella in Enteropathogenic *Escherichia coli* Requires the Presence of a Functional Type III Secretion System

**DOI:** 10.3390/ijms232213705

**Published:** 2022-11-08

**Authors:** Jorge Soria-Bustos, Zeus Saldaña-Ahuactzi, Partha Samadder, Jorge A. Yañez-Santos, Ygnacio Martínez Laguna, María L. Cedillo-Ramírez, Jorge A. Girón

**Affiliations:** 1 Escuela de Biología, Benemérita Universidad Autónoma de Puebla, Puebla 72410, Mexico; 2 Department of Immunobiology, University of Arizona, Tucson, AZ 85721, USA; 3 Centro de Investigación en Ciencias Microbiológicas, Benemérita Universidad Autónoma de Puebla, Puebla 72410, Mexico; 4 Facultad de Estomatología, Benemérita Universidad Autónoma de Puebla, Puebla 72410, Mexico; 5 Centro de Detección Biomolecular, Benemérita Universidad Autónoma de Puebla, Puebla 72410, Mexico

**Keywords:** T3SS, virulence, flagella, enteropathogenic, *Escherichia coli*

## Abstract

In enteropathogenic *Escherichia coli* (EPEC), the production of flagella and the type III secretion system (T3SS) is activated in the presence of host cultured epithelial cells. The goal of this study was to investigate the relationship between expression of flagella and the T3SS. Mutants deficient in assembling T3SS basal and translocon components (Δ*espA*, Δ*espB*, Δ*espD*, Δ*escC*, Δ*escN*, and Δ*escV*), and in secreting effector molecules (Δ*sepD* and Δ*sepL*) were tested for flagella production under several growth conditions. The Δ*espA* mutant did not produce flagella in any condition tested, although *fliC* was transcribed. The remaining mutants produced different levels of flagella upon growth in LB or in the presence of cells but were significantly diminished in flagella production after growth in Dulbecco’s minimal essential medium. We also investigated the role of virulence and global regulator genes in expression of flagella. The Δ*qseB* and Δ*qseC* mutants produced abundant flagella only when growing in LB and in the presence of HeLa cells, indicating that QseB and QseC act as negative regulators of *fliC* transcription. The Δ*grlR*, Δ*perA*, Δ*ler*, Δ*hns*, and Δ*fis* mutants produced low levels of flagella, suggesting these regulators are activators of *fliC* expression. These data suggest that the presence of an intact T3SS is required for assembly of flagella highlighting the existence in EPEC of a cross-talk between these two virulence-associated T3SSs.

## 1. Introduction

Flagella are multi-purpose structures that provide bacteria with the ability to swim, and they are also associated with other virulence-associated properties in a wide range of pathogenic bacteria [1,2]. These properties include adherence, invasion, colonization, hemagglutination, biofilm formation, binding to host proteins, secretion of flagellar proteins, induction of Toll-like receptor 5-dependent proinflammatory responses, and translocation of virulence molecules [3,4,5,6,7,8]. The mechanisms that dictate flagella assembly, chemotaxis, and motility are remarkably complex, involving many genetic elements and regulatory networks [2]. The components of the flagella apparatus of *Escherichia coli* and *Salmonella enterica* serovar Typhimurium are encoded in at least 50 genes comprised in 17 operons, which are regulated by the master regulon *flhDC* [2,9,10]

An emerging concept in the biology of bacteria that infect plants, insects, and animals is the presence of a specialized secretion machinery called the type III secretion system (T3SS) (also called injectisome, secreton, or translocon), which is devoted to the secretion and injection of effector molecules into the host target cell [11,12,13]. These effectors have a wide range of biological activities that enable the bacteria to successfully exploit signaling mechanisms that lead to cytoskeleton reorganization, induce apoptosis, modulate the cell cycle, invade cells, target tight junctions, suppress the host innate immunity or down-regulate pro-inflammatory responses in the human host. The basal bodies of the T3SS and the flagellin-export apparatus devoted to build up flagella show striking structural similarities [14,15]. The export of flagellins and build-up of flagella also occurs in a T3SS-like mechanism and, thus, the flagella export system is also considered a T3SS machinery. It has been proposed that both secretion systems have jointly evolved to guarantee bacterial survival and pathogenicity within their hosts.

Complex regulatory genetic networks exist that rigorously and independently control flagellar or T3SS gene expression in response to specific environmental conditions to optimize virulence [16,17,18,19]. In enteropathogenic *E. coli* (EPEC), the T3SS is regulated by the plasmid-encoded regulator (Per), which is encoded on the EAF plasmid that also codes for the bundle-forming pilus (BFP) [20,21]. The latter is associated with the formation of clusters of microcolonies on the surface of epithelial cells, a pattern called localized adherence (LA) [22].

It was reported that *Pseudomonas aeruginosa* deficient in production of flagella and motility produced increased levels of T3SS needles and secreted abundant effectors in comparison to the wild-type strain [23,24]. In *S. enterica* serovar Typhi, a positive cross-talk exists between the flagella and the SPI-1 regulon that encodes a T3SS [25]. In *Yersinia enterocolitica*, the master regulatory proteins FlhCD exert a negative control on the T3SS Yop regulon [26]. Hence, it would appear that in some pathogenic bacteria, the regulation of the production and function of flagella and the T3SS are inter-related; however, the molecular processes that couple these T3SSs remain to be defined.

The flagella of EPEC have adhesive properties, they bind to mucins, extracellular matrix proteins such as collagen, intestinal mucus, and host epithelial cells [6,27]. The production of EPEC’s flagella is triggered by the presence of cultured epithelial cells [6,19]. We have previously reported that strains mutated in T3SS-associated genes (*escN*, *eae*, *espA*, *espB*, *tir*, and *espD*) were deficient in expression of flagella and motility when growing in Dulbecco’s minimal essential medium (DMEM) but not in Luria–Bertani (LB) broth [6]. Moreover, flagellation and motility in EPEC E2348/69 could be restored upon growth in the presence of HeLa cells or supernatants thereof [6,19]. The apparent requirement of a functional T3SS for adequate synthesis and function of flagella in EPEC, suggest the existence of a molecular feedback between the flagellar regulon and T3SS genes. The virulence of EPEC is under the control of virulence regulators (Per, Ler, GrlR, and GrlA) and a myriad of global regulators such as IHF, BipA, H-NS, RpoS, Fis, and QseBC [28,29,30,31,32]. Ler is encoded by the locus of enterocyte effacement (LEE) pathogenicity island, which harbors the genes required for assembly of the T3SS [33]. The GrlA and GrlR proteins are positive and negative LEE-encoded regulators of LEE genes in EPEC, enterohemorrhagic *E. coli* O157:H7 (EHEC) and *Citrobacter rodentium*, respectively [34,35,36]. In EHEC, GrlR was shown to down-regulate flagella expression [34]. In this paper, we provide a body of compelling evidence that strongly establishes an intimate cross talk between flagella and the T3SS of EPEC. An intact T3SS is required for adequate flagella production and display of motility. The synchronized activation of T3SS and flagella production would presumably provide a potential benefit to the bacteria for efficient colonization of the human gut mucosa.

## 2. Results

### 2.1. The EspA Fiber Is Required for Flagella Assembly

**Figure 1:**
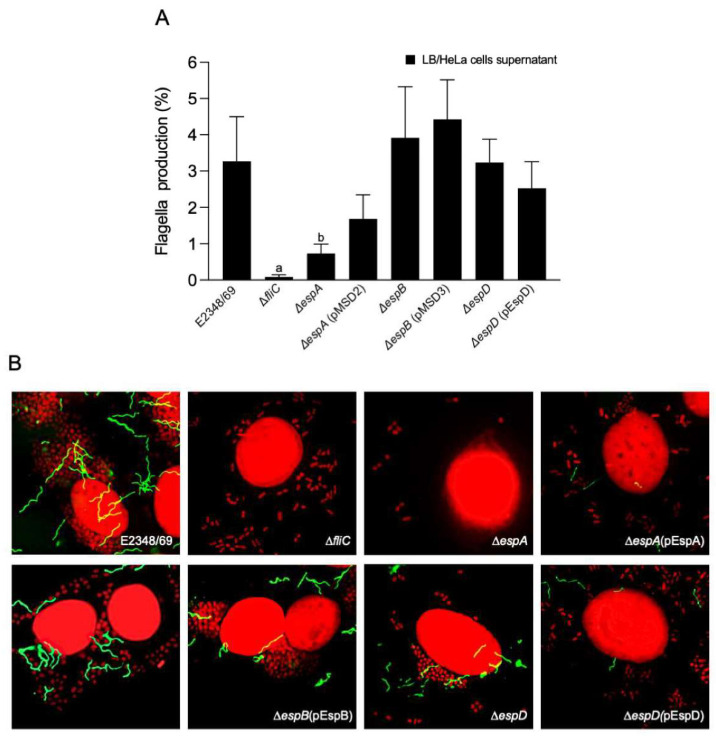
Production of flagella by EPEC strains defective in T3SS. (**A**) Flow cytometry was used to quantitatively determine flagella production. LB-grown bacteria were used to infect HeLa cells for 3 h after which the supernatants were collected and analyzed by flow cytometry using rabbit anti-H6 antibodies and anti-rabbit IgG Alexa–fluor 488. The data shown are the mean of three experiments performed in triplicate on different days. Letters a and b show statistical significance of the mutants tested with respect to E2348/69 (one-way ANOVA test, followed by Tukey’s multiple comparison), a = *p* < 0.001; b = *p* < 0.01. (**B**) Flagella produced by the indicated LB-grown bacterial strains adhering to HeLa cells were visualized by immunofluorescence using rabbit anti-H6 flagella antibodies and anti-rabbit IgG Alexa-fluor 488. Immunofluorescence images were taken at 60×.

In the course of studies of EPEC flagella, we noted that strains carrying mutations in genes associated with assembly of the T3SS apparatus (*espA*, *espB*, and *espD*) or in secretion of T3 effectors (*escN*) were deficient in flagella production and motility [6]. Moreover, it was reported that mutants in *espA* or *espB* genes were unable to adhere efficiently (11% and 36% reduction, respectively) to host cells due to the inability to inject the translocated-intimin receptor Tir [37,38]. It is well known that the expression of flagella and the T3SS in EPEC is influenced by nutritional and host factors [6,19]. In this study, we wanted to further understand the functional relationship between these two T3SS machineries of EPEC. In the first set of experiments, EPEC E2348/69 isogenic mutants in genes that code for the EspA, EspB, and EspD proteins were cultured overnight in LB broth at 37 °C. HeLa cell monolayers were infected with 10 μL of the LB cultures and incubated for 3 h as described in Materials and Methods. After incubation, the supernatants were collected and the bacterial concentration was adjusted to an optical density at 600 nm (OD_600_) of 1.1 before analysis by flow cytometry using rabbit polyclonal anti-H6 antibodies as the probe. In parallel, the infected HeLa cell monolayers were washed and analyzed by immunofluorescence for the presence of flagella on bacteria adhering to these cells. The flow cytometry data showed that the Δ*espA* mutant produced 77.6% fewer flagella than the wild-type strain (*p* < 0.01) (Figure 1A).

In contrast, the Δ*espB* and Δ*espD* mutants showed no statistical differences in flagella production with respect to E2348/69. As expected, the Δ*fliC* mutant showed no flagella. Interestingly, when these strains were analyzed by immunofluorescence microscopy for flagella production, we found that the Δ*espB* and Δ*espD* mutants produced detectably fewer flagella than the wild-type strain, while Δ*espA* and Δ*fliC* mutants displayed no flagella (Figure 1B and Table 1). The presence of flagella correlates, of course, with the amount of host-cell associated bacteria. The poor adherence observed correlates with previous reports and also explains, in part, the low number of flagella [37,38,39]. Complementation in *trans* of the mutants restored flagella production (Figure 1).

**Figure 2:**
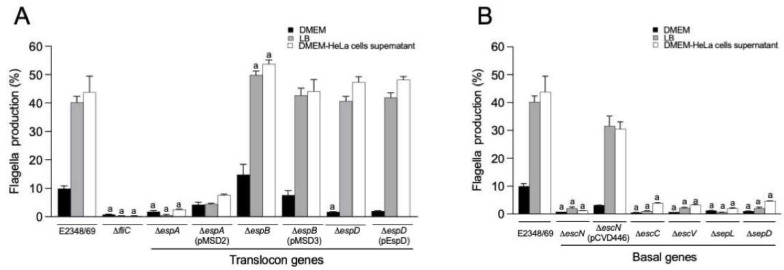
Growth conditions affect production of flagella by T3SS mutants. (**A**,**B**) Flow cytometry was used to quantitatively determine the production of flagella by translocon-associated Δ*espA*, Δ*espB*, and Δ*espD* mutants and the basal-body Δ*escN*, Δ*escC*, Δ*escV*, Δ*sepL*, and Δ*sepL* mutants, respectively. For these experiments the strains were grown as described in Materials and Methods. The data shown are the mean of three experiments performed in triplicate on different days. Letter a shows the statistical significance of the mutants tested with respect to the E2348/69 strain grown in each condition (one-way ANOVA test, followed by Tukey’s multiple comparison), a = *p* < 0.001.

In the next set of experiments, we wanted to know if the nature of the growth medium growth used to obtain the inoculum was a factor in flagella production. Thus, the wild-type strain and the Δ*espA*, Δ*espB*, and Δ*espD* mutants were grown in DMEM, LB, and with HeLa cell monolayers for 3 h. LB- and DMEM-grown bacteria, and bacteria recovered from the supernatants of infected cells were analyzed for flagella production by flow cytometry. We found that E2348/69 growing in DMEM produced significantly less flagella (~75% reduction) than when growing in LB or in the presence of HeLa cells (*p* < 0.001) (Figure 2A). A similar effect was observed with the Δ*espB* (~70% reduction) and Δ*espD* (~96% reduction) mutants (*p* < 0.001) (Figure 2A). However, increased levels of flagella were detected in the Δ*espB* mutant, compared with the wild-type strain in the three growth conditions tested (*p* < 0.001). No statistical difference was found between mutants and the complemented strains in all conditions evaluated. The Δ*fliC* mutant used as negative control showed no detectable flagella in any of the conditions tested. The Δ*espA* mutant was deficient in flagella production in all the conditions examined (*p* < 0.001). The Δ*espA* complemented strain produced more flagella than the mutant. These results were confirmed by immunoblotting (Supplementary Figure S1). In sum, these data suggest that the growth medium and host factors influence flagella production and that the presence of an intact EspA fiber is required for the assembly of flagella.

### 2.2. T3SS Basal Components Are Required for Flagella Production

**Figure 3:**
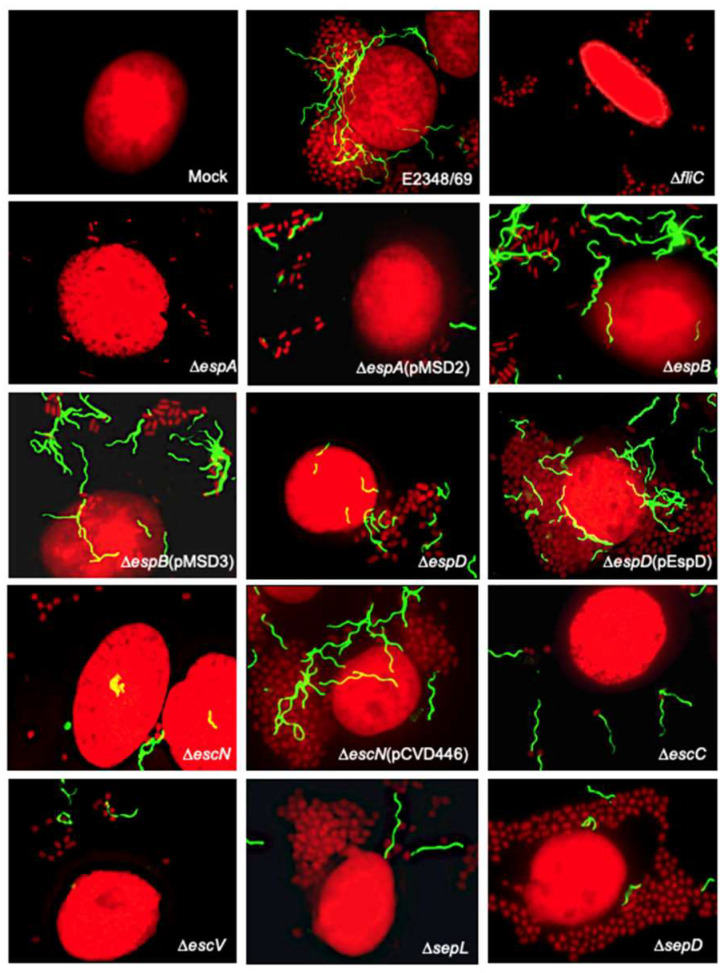
Visualization of flagella by DMEM-grown EPEC strains adhering to HeLa cells. Flagella (green) produced by the indicated bacterial strains adhering to HeLa cells were visualized by immunofluorescence using rabbit anti-H6 flagella antibodies and anti-rabbit IgG Alexa-fluor 488. The cellular and bacterial DNA was stained with propidium iodide (red). Immunofluorescence images were taken at 60×.

Next, we inquired about the requirement of other structural basal components of the T3SS translocon in flagella assembly. We used isogenic mutants unable to produce EscN (ATPase that provides energy for secretion and translocation), EscC and EscV (basal T3SS components), and SepD and SepL, which regulate the secretion of effector molecules. A mutation in *escC* or *escV* genes, render the bacteria unable to assemble a T3SS structure on the surface and to adhere efficiently to host cells [35]. The Δ*sepD* and Δ*sepL* mutants are affected in secretion of effectors and in adherence to cultured cells [40] and a mutation in *escN* results in the lack of secretion of effectors and poor cell adherence [11,41]. In contrast to the wild type, we found that the Δ*escN*, Δ*escC*, Δ*escV*, Δ*sepL*, and Δ*sepD* strains produced significantly lower levels of flagella after growth in LB or DMEM or in the presence of HeLa cells (*p* < 0.001) (Figure 2B). However, when adhering to HeLa cells, only a few flagella filaments were detected in the Δ*escN*, Δ*escC*, Δ*escV*, Δ*sepD*, and Δ*sepL* mutants (Figure 3 and Table 1). These data are in agreement with the flow cytometry data. These strains were poorly adherent at 3 h of incubation, which can also explain the poor levels of flagella seen by immunofluorescence. The Δ*escN* mutant complemented in *trans* with *escN* on plasmid pCVD446, showed many more flagella and more adhering bacteria than the Δ*escN* mutant, as expected. No complemented strains were available for the Δ*escC*, Δ*escV*, Δ*sepD*, and Δ*sepL* mutants. These data indicate that when the T3SS apparatus is missing or non-functional there is no flagella assembly.

### 2.3. Transcriptional Analysis of fliC in T3SS Mutants

**Figure 4:**
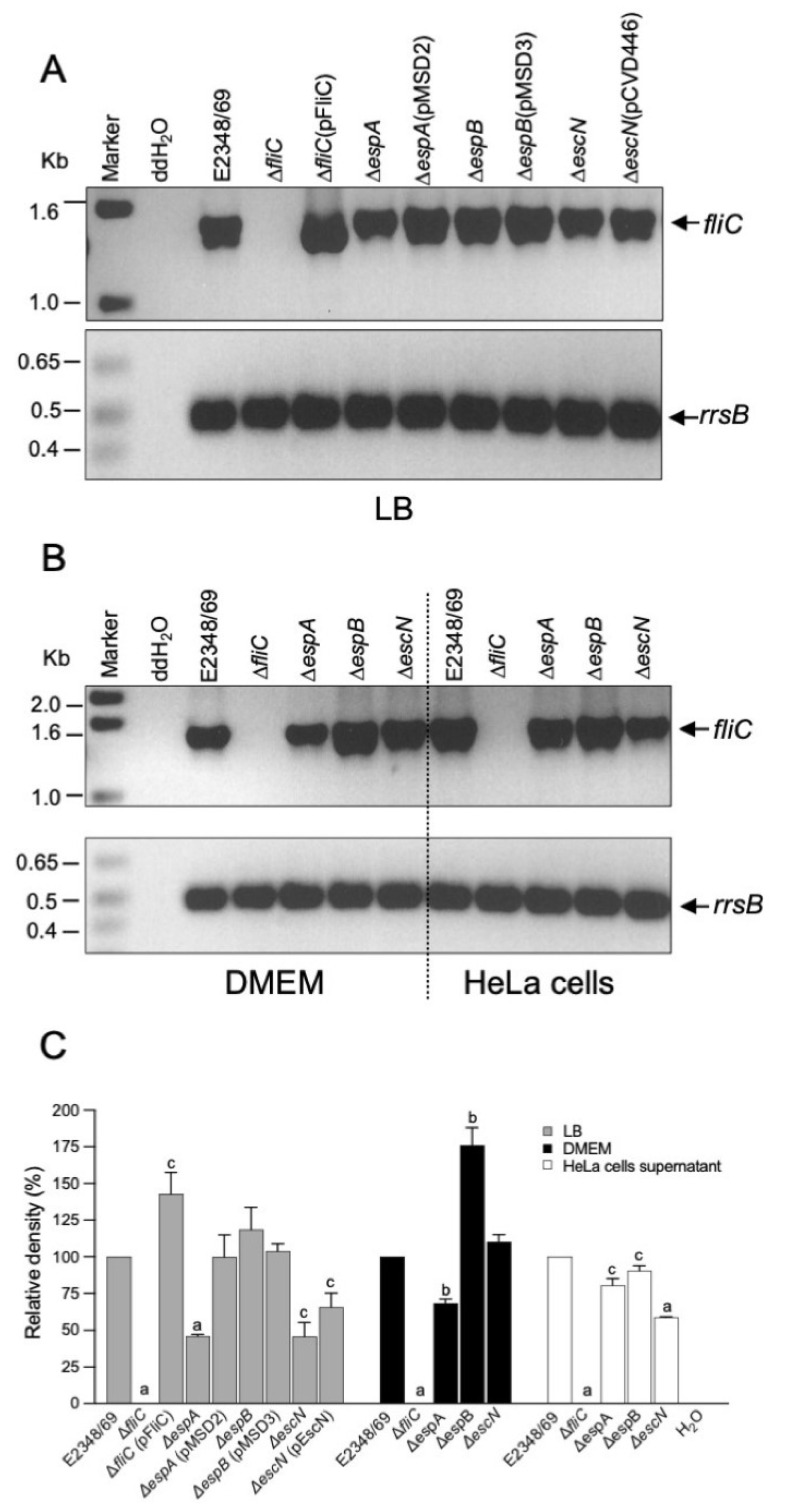
Transcription analysis of *fliC* in the different EPEC strains. (**A**,**B**) RT-PCR to determine expression of *fliC* in the indicated strains after growth in LB, DMEM, and in the presence of epithelial cells. (**C**) Densitometric analysis derived from the mRNA agarose gel electrophoresis (**A**) and (**B**) showing quantitative *fliC* expression in EPEC E2348/69 and its derivative mutants. The quantitative data are the mean of three different measurements. Letters a, b, and c show statistical significance of the mutants tested with respect to E2348/69 grown in each condition (one-sample *t* test), a = *p* < 0.001; b = *p* < 0.01; c = *p* < 0.05. The Δ*fliC* mutant was used as the negative control and detection of *rrsB* was used as a loading control.

Given that most of the mutants in T3SS genes lacked or were deficient in flagella production we monitored *fliC* expression by RT-PCR to determine if the mutations were affected at the level of *fliC* transcription. All of the strains seemed to express *fliC* (Figure 4A,B). However, when the different strains were analyzed by a semi-quantitative digital analysis, a significant reduction in the expression of *fliC* was detected in the Δ*espA and* Δ*escN* mutants when they were grown in LB (~55% reduction) or incubated with HeLa cells (20% and 42% reduction, respectively) with respect to the E2348/69 strain (Figure 4C). Interestingly, incremental *fliC* expression was detected in the Δ*espB* mutant in LB (18%) and DMEM (79%) (*p* < 0.01). In all, these results suggest that the repression of flagella seen in the T3SS mutants is not at the transcriptional level, but at the post-transcriptional level.

### 2.4. Regulation of Flagella by Virulence and Global Regulators

**Figure 5:**
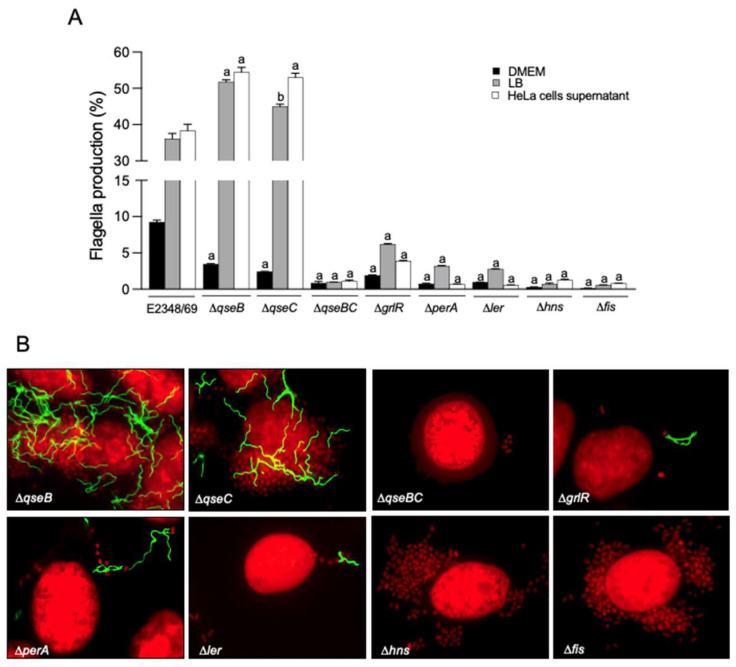
Influence of virulence and global regulators in the production of flagella. (**A**) Flow cytometry and (**B**) immunofluorescence were performed to determine the production of flagella by the indicated mutants. Immunofluorescence images were taken at 60×. Data shown are the mean of three experiments performed in triplicate on different days. Letter a shows the statistical significance of the mutants tested with respect to E2348/69 grown in each condition (one-way ANOVA test, followed by Tukey’s multiple comparison), a = *p* < 0.001; b = *p* < 0.01.

To further elucidate if the hypothetic cross-talk between flagella and T3SS was dependent on virulence or global regulators, we determined flagella production in Δ*qseB*, Δ*qseC*, and Δ*qseBC* quorum-sensing mutants, Δ*grlR*, Δ*perA*, Δ*ler*, Δ*hns*, and Δ*fis*. While H-NS and Fis are global regulators of house-keeping and virulence genes [28,42], the two-component system QseBC regulates quorum sensing, virulence factors, and the expression of flagella and motility in EHEC [31]. Interestingly, the Δ*qseB* and Δ*qseC* mutants produced abundant flagella when growing in LB (*p* < 0.001 and *p* < 0.01, respectively) and in the presence of HeLa cells (*p* < 0.001), but were significantly reduced in flagella synthesis in DMEM (Figure 5A). These data indicate that these quorum sensing regulators function also as repressors of flagella transcription. In contrast, the remaining mutants analyzed showed poor levels of flagella production in all the conditions tested, which suggests that PerA, H-NS, and Fis activate flagella production (Figure 5). The immunofluorescence data shown correlated with the flow cytometry data (Figure 5B and Table 1).

### 2.5. Motility of EPEC Strains

**Figure 6:**
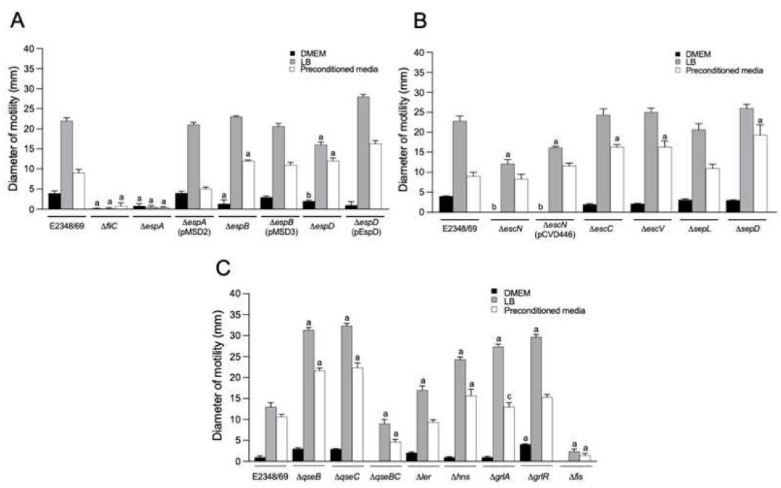
Motility of strains mutated in virulence and global regulators. (**A**–**C**) Motility of the indicated strains grown in LB, DMEM and preconditioned DMEM containing 0.3% agar. The data shown are the mean of three experiments performed in triplicate on different days. Letters a, b, or c show the statistical significance of the mutants tested with respect to E2348/69 grown in each condition (one-way ANOVA test, followed by Tukey’s multiple comparison), a = *p* < 0.001; b = *p* < 0.01; c = *p* < 0.05.

To correlate the presence or absence of flagella in the EPEC strains with their ability to swim, we assayed all of the mutants in T3SS genes and regulators, in motility medium employing as base LB, DMEM, or preconditioned medium (which is a filtered supernatant of HeLa cell cultures) with 0.3% agar. In agreement with the data shown above, the Δ*espA* mutant was non-motile in all the conditions tested (*p* < 0.001), highlighting the requirement of an intact EspA fiber for production of flagella and motility. Generally, growth in DMEM significantly repressed motility in the wild-type strain and all of the mutants (Figure 6 and Table 1). However, except for the Δ*espA*, Δ*espD*, Δ*escN*, Δ*qseBC*, and Δ*fis* mutants, the rest of the strains displayed wild-type-like motility when growing in LB medium (Figure 6). When motility was assayed in pre-conditioned soft agar medium, motility was reduced in all the strains in comparison to LB motility medium (Table 1).

### 2.6. fliC and motB Mutants Are Not Affected in T3 Protein Secretion

**Figure 7:**
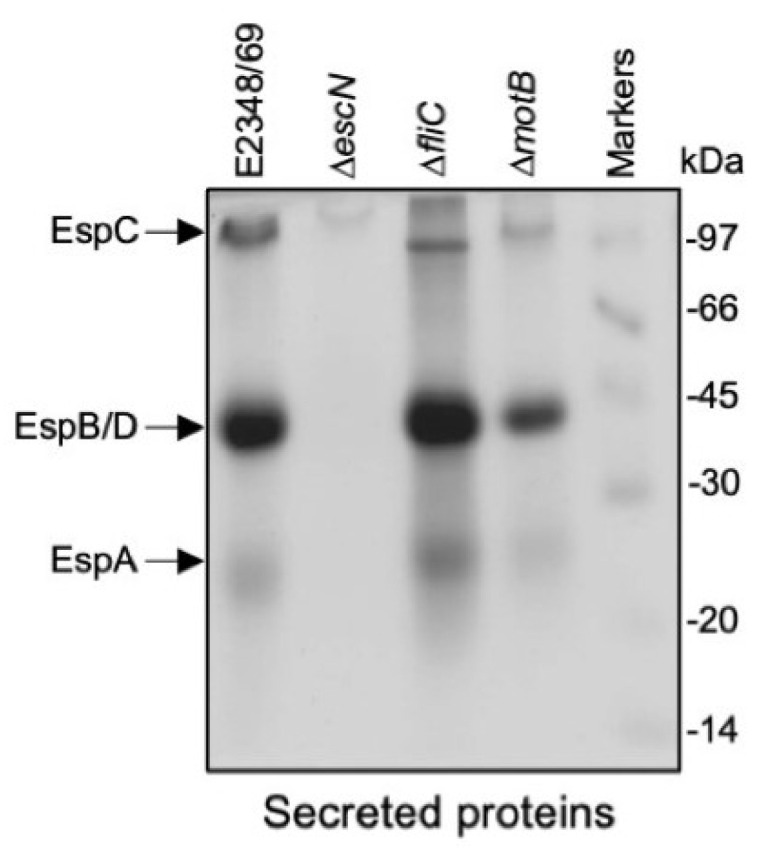
Analysis of T3 secreted proteins in wild type E2348/69 and derivative mutants. Filtered and concentrated supernatants from bacteria grown in DMEM were electrophoresed in 14% SDS–PAGE gels. The Δ*escN* mutant (unable to assemble a T3SS and secrete proteins) was used as negative control.

Flagella mutants of *P. aeruginosa* PAO1 produce more T3SS needle structures than the wild-type strain [23,24]. Conversely, EPEC T3SS mutants produce little to no flagella (Figure 1, Figure 2 and Figure 3). The data above indicate a strong correlation between expression of the T3SS and flagella in EPEC. We were interested in knowing if T3 protein secretion was affected in mutants possessing T3SS but lacking the flagellin gene (Δ*fliC* mutant) or a Δ*motB* mutant unable to rotate flagella. We sought the known secreted proteins Tir, EspB, EspD, and EspA, in DMEM cultures as previously described [43]. Further, actin condensation was determined in HeLa cells as an indication of T3SS function in host cells and consequently of the production of attaching and effacing lesions. As shown in Figure 7, secretion of the most abundant secreted proteins Tir, EspB/D, and EspA was not affected in the Δ*fliC* and Δ*motB* mutants as compared to the wild-type strain. No Esps were found in the Δ*escN* mutant used as negative control. These results correlated with the FAS assay results, which showed that, except for the Δ*escN* mutant, the remaining strains were able to recruit actin beneath the adhering bacteria. Thus, the T3SS was not affected in the Δ*fliC* and Δ*motB* mutants (Table 1).

## 3. Discussion

Flagella are surface appendages that propel bacteria towards nutrient-rich environments or help bacteria escape from environmental foes [2]. Motility is considered beneficial for bacterial pathogens for colonization of host mucosal surfaces generally bathed by a protective mucus gel. Many illness-causing bacteria inject numerous effector proteins, which have specific targets in host cells as part of the pathogenic scheme [39,44,45]. The injection of these effectors is achieved by a needle-like secretion machinery called the T3SS. Structural analysis of the basal bodies of the T3SS and flagella revealed striking similarities. Both virulence-associated machineries are strictly regulated by specific genetic elements and regulatory networks in response to specific environmental conditions [2,16,17,18,46,47]. Virulence-devoted regulators are also known to modulate flagellation and the production of the injectisome according to the host’s niche to ensure that they are most efficient during interaction with the host cell. In addition, in pathogenic bacteria such as *S. enterica*, *Y. enterocolitica*, and *P. aeruginosa*, the flagellar regulon exerts a negative effect over the production of T3SS. Conversely, previously published data showed that EPEC mutants lacking or defective in T3SS function are also defective in flagella production and motility when growing in DMEM [6]. Thus, it is apparent and conceivable that a molecular cross-talk exists between flagella and the T3SS, which, in this study, we sought to address. We began by studying the production of flagella in mutants (Δ*espA*, Δ*espB*, and Δ*espD*) defective in assembling the secretory needle, which is built up of polymerized EspA, at the tip of which, the EspB and EspD proteins sit and form a pore through the host cell membrane. First, we analyzed flagella production in bacteria after 3 h-incubation with HeLa cells, in which the inoculum for these infections was originated from LB cultures. The Δ*espA* mutant was unable to produce flagella and to swim while the Δ*espB* and Δ*espD* mutants produced a similar number of flagella to the wild-type strain. The complementation in *trans* of the Δ*espA* mutant restored flagellation. We also inquired about the role of growth media (LB, DMEM) as well as the presence of HeLa cells in expression of *fliC* and production of flagella using DMEM cultures as inocula. Growth in either LB or DMEM repressed flagella production in the Δ*espA* mutant. However, the Δ*espB* and Δ*espD* mutants produced flagella after growth in LB and in the presence of HeLa cells. The data strongly suggest that an intact EspA needle is required for flagella to be optimally produced. *fliC* transcription in the Δ*espA* mutant was reduced by 50% with respect to E2348/69 when growing in LB, 25% in DMEM, and 20% in the presence of HeLa cells. The presence of *fliC* mRNA in this mutant does not account for the demonstrated lack of flagella. Thus, it is possible that other post-transcriptional or translational effects are responsible for the absence of flagella in the Δ*espA* mutant.

Next, we questioned whether basal components of the T3SS apparatus (EscN, EscC, EscV) or regulators of protein secretion (SepL and SepD) are also needed for flagella production. Interestingly, none of these mutants produced significant amounts of flagella under the growth conditions tested. A few flagella filaments were seen associated with the bacteria adhering to HeLa cells.

Genes that regulate EPEC virulence factors such as PerA, Ler, and GrlR as well as global regulators of house-keeping genes (QseB, QseC, H-NS, and Fis) have been reported to also be involved in regulatory control of flagella or T3SS [34,48]. We inquired about the interception of these regulators with flagella production. None of the mutants in these regulators produced flagella upon growth in DMEM. However, the quorum sensing mutants produced high levels of flagella upon growth in LB and in the presence of HeLa cells. Clearly, QseB and QseC act as negative activators of flagella transcription. In contrast, the remaining regulator mutants analyzed showed low or undetectable levels of flagella production (Figure 5), which suggest that LEE-encoded regulators, PerA, H-NS, and Fis activate flagella production in EPEC.

Previous work has shown that flagella mutants of *P. aeruginosa* produce an increased amount of T3SS needles [23], suggesting the existence in this organism of a cross-talk between flagella and T3SS. Thus, we tested if strains Δ*fliC* and Δ*motB* mutants of E2348/69 were deficient in secretion of T3S proteins or in producing attaching and effacing (A/E) lesions. No differences in secreted proteins between the wild-type strain and flagella mutants were seen and they were still able to produce A/E lesions suggesting that in contrast to *P. aeruginosa*, EPEC flagella or motility mutants are not affected in T3SS. In sum, our data highlights the need for an intact T3SS needle in the production of flagella. The precise molecular mechanism of the cross-talk between the two T3SSs remains unknown and warrants further investigation. It is conceivable that the requirement of a functional T3SS on the surface of bacteria for production of flagella is an advantage for the bacterial pathogen in its interaction with the host and genesis of disease.

## 4. Materials and Methods

### 4.1. Bacterial Strains, Plasmids, and Culture Conditions

Bacterial strains employed are listed in Table 2 and they were grown overnight with shaking either in Luria–Bertani (LB) broth (Sigma, St. Louis, MO, USA), low-glucose Dulbecco’s minimal essential medium (DMEM) (Gibco), or in the presence of HeLa cells at 37 °C.

### 4.2. Construction of Isogenic Mutants

Deletion mutants were generated by the λ Red recombinase method previously described [56]. Each purified PCR product was electroporated into competent EPEC E2348/69 carrying the λ Red recombinase helper plasmid PKD46, whose expression was induced by adding L-(+)-arabinose (Sigma, St. Louis, MO, USA) at a final concentration of 1.0%. PCR fragments containing the specific sequences flanking either kanamycin or chloramphenicol cassette, were generated using gene-specific primers. PKD4 and PKD3 were used as a template, respectively. To complement the mutations, different plasmids were used (Table 1). Different constructs were generated by cloning a specific PCR product containing the corresponding region of interest into the specific plasmid previously digested.

### 4.3. Flow Cytometry

To quantify the production of flagella, strains were cultured overnight in DMEM or LB broth at 37 °C. HeLa cell monolayers were infected with 10 μL of the DMEM or LB cultures and incubated for 3 h. After incubation, the supernatants were collected and the bacterial concentration was adjusted to an OD_600_ of 1.1 before analysis by flow cytometry. Then, 45-µL aliquots were incubated for 1 h on ice with 25 µL of rabbit polyclonal anti-H6 antibody (1:1000). After three gentle washes with PBS, the bacteria were suspended in 25 µL of a dilution of goat anti-rabbit IgG (H+L) Alexa Fluor conjugate (Invitrogen, Carlsbad, CA, USA). After 1-h incubation at 4 °C, the bacteria were washed again and resuspended in 800-µL final volume of PBS. For the analysis, the bacteria were labeled with 5 µL of a propidium iodide solution (Sigma, St. Louis, MO, USA), which was visualized through a 42-nm band pass centered at 585 nm. These experiments were performed three times in triplicate. The FITC fluorescence emission was collected through a 30-nm band-pass filter centered at 530 nm in which 50,000 events were measured. As negative controls, reactions with preimmune serum and the *fliC* mutant were included [48].

### 4.4. Adherence to Epithelial Cells and Detection of Flagella by Immunofluorescence

HeLa cells were seeded onto polystyrene 24-well plates (CELLSTAR) containing glass coverslips and propagated at 37 °C under a 5% CO_2_ atmosphere, as previously described [6]. Strains were cultured overnight in DMEM or LB broth at 37 °C. HeLa cell monolayers were infected with ~10^7^ of LB- or DMEM-grown bacteria and incubated for 3 h. After incubation, the cells were washed with PBS to remove unbound bacteria and the samples were fixed with 2% formalin/PBS for immunofluorescence. Primary rabbit anti-H6 antibodies were added for 1 h at 1:3000 dilution in 10% normal horse serum. After washing, the cells were incubated for 1 h with secondary anti-rabbit IgG Alexa fluor-conjugated antibodies diluted 1:3000. The cells were washed extensively and mounted in glycerol-PBS and visualized under a UV light using a Zeiss Axiolab microscope. FITC-labelled phalloidin (Sigma, St. Louis, MO, USA) was used in the FAS assay to detect A/E lesions as previously described [57]. Immunofluorescence images were taken with a 60× objective.

### 4.5. Immunoblotting

Production of flagellin was monitored by immunoblotting using DMEM-grown bacterial cultures and adjusted to an OD_600_ of 1.1. Equal numbers of bacteria were used to prepare whole-cells extracts by denaturation in SDS-PAGE sample buffer and separated in 14% SDS-PAGE gels. Proteins were electroblotted onto PVDF membranes and reacted for 1 h with rabbit anti-flagella H6 antibodies (1:3000) [6] and secondary goat anti-rabbit IgG conjugated to horseradish peroxidase (1:20,000) (Sigma, St. Louis, MO, USA) [6]. Detection of DnaK with anti-DnaK antibody was used as a loading control.

### 4.6. RT-PCR Assay

Total RNA was extracted from bacterial cultures using Trizol Reagent (Invitrogen, Carlsbad, CA, USA) following the manufacturer’s guidelines. Prior to RT-PCR, 2 μg of total RNA were treated with RQ1 RNAse-free DNase, according to the manufacturer’s protocol. Specific transcripts were amplified using a one-step RT-PCR kit (Qiagen, Hilden, Germany) and 0.1 μg/μL of total RNA as template. 16S RNA (*rrsB*) was used as a loading control. The semi-quantitative measurement of PCR amplicons was performed by digital analysis of the RT-PCR electrophoresed gels as previously described [58,59]. The relative density for *fliC* amplicons from the different mutants was expressed in percentage, and compared to the wild-type strain.

### 4.7. Motility Assay

Motility assays were performed in 0.3% agar plates containing LB, DMEM, or preconditioned medium (which was a filtered supernatant of HeLa cell cultures). Briefly, the agar was spiked with overnight cultures and incubated at 37 °C. The motility was assessed by examining the radius of opacity as a result of bacterial swimming away from the point of inoculation after 16 h of incubation.

### 4.8. Statistical Analysis

All quantitative data were the averages of three independent experiments performed in triplicate. Statistical significance was determined by comparing flagella production and the relative density of *fliC* expression of the different mutants with respect to the E2348/69 strain grown in each condition employing the one-way ANOVA test followed by Tukey’s multiple comparison test and the one-sample *t* test, respectively. A *p*-value of ≤0.05 was considered statistically significant. The GraphPad Prism 9 software (GraphPad, San Diego, CA, USA) was used for the statistical analysis.

## Figures and Tables

**Table 1:** Fluorescent actin-staining (FAS) assay, adherence, flagella presence, and motility of E2348/69 and derived mutants.

Strains	Adherence		Flagella		Motility		FAS	
	LB	DMEM	LB	DMEM	LB	DMEM	DMEM	PM
E2348/69	LA	LA	+++	++	+++	+	+	++
E2348/69Δ*flic*	++	+	-	-	-	+	-	-
E2348/69Δ*espA*	+	+	-	-	-	ND	-	-
E2348/69Δ*espA*(pEspA)	++	++	+	+	+++	ND	+	+
E2348/69Δ*espB*	+	+	+	++	+++	ND	+	++
E2348/69Δ*espB*(pEspB)	++	+	++	++	+++	ND	+	++
E2348/69Δ*espD*	+	+	+	+	++	ND	+	++
E2348/69Δ*espD*(pEspD)	++	++	+	++	+++	ND	+	++
E2348/69Δ*escN*	ND	+	ND	+	++	-	-	++
E2348/69Δ*escN*(pEscN)	ND	LA	ND	++	+++	ND	-	++
E2348/69Δ*escC*	ND	+	ND	+	+++	ND	+	++
E2348/69Δ*escV*	ND	+	ND	+	+++	ND	+	++
E2348/69Δ*sepL*	ND	++	ND	+	+++	ND	+	++
E2348/69Δ*sepD*	ND	++	ND	+	+++	ND	+	++
E2348/69Δ*qseB*	ND	++	ND	+++	+++	ND	+	+++
E2348/69Δ*qseC*	ND	++	ND	++	+++	ND	+	+++
E2348/69Δ*qseBC*	ND	+	ND	-	-	ND	++	+
E2348/69Δ*perA*	ND	+	ND	+	ND	ND	ND	ND
E2348/69∆*grlA*	ND	ND	ND	ND	+++	ND	+	++
E2348/69∆*grlR*	ND	+	ND	+	+++	ND	+	++
E2348/69∆*ler*	ND	+	ND	+	+++	ND	+	++
E2348/69∆*hns*	ND	++	ND	-	+++	ND	+	++
E2348/69∆*fis*	ND	++	ND	-	+	ND	-	+
E2348/69Δ*motB*	ND	ND	ND	ND	ND	+	ND	ND

PM, preconditioned media; LA, localized adherence; ND, not done; (-) = non-adherent, no flagella or non-motile; (+) = weakly-adherent, poor flagella production or weakly-motile; (++) = moderately-adherent, moderately flagellate or moderately-motile; (+++) = highly-adherent, highly flagellate or highly-motile.

**Table 2:** Strains and plasmids used in this study.

Strains	Notes	Reference
E2348/69	EPEC (O127:H6) Wild type	[49]
E2348/69Δ*fliC*	*fliC*::km mutant	[6]
E2348/69Δ*espA*	*espA*::km mutant	[6]
E2348/69Δ*espB*	*espB*::km mutant	[6]
E2348/69Δ*espD*	*espD*:km mutant	[6]
E2348/69Δ*escN*	*escN*::km mutant	[6]
E2348/69Δ*escC*	*escC*::km mutant	[41]
E2348/69Δ*escV*	*escV*::km mutant	This study
E2348/69Δ*sepL*	*sepL*::km mutant	This study
E2348/69Δ*sepD*	*sepD*::km mutant	This study
E2348/69Δ*qseB*	*qseB*::km mutant	[50]
E2348/69Δ*qseC*	*qseC*::cm mutant	This study
E2348/69Δ*qseBC*	*qseBC*::cm mutant	This study
E2348/69Δ*perA*	*perA*::km mutant	[51]
E2348/69∆*grlA*	*grlA*::km mutant	[51]
E2348/69∆*grlR*	*grlR*::km mutant	[52]
E2348/69∆*ler*	*ler*::km mutant	[51]
E2348/69∆*hns*	*hns*::km mutant	Bustamante et al., unpublished
E2348/69∆*fis*	*fis*::km mutant	[53]
E2348/69Δ*motB*	*motB*::cm mutant	[6]
**Plasmids**		
pKD46	Red recombinase system plasmid	
pKD4	Kanamycin cassette template plasmid	
pKD3	Chloramphenicol cassette template plasmid	
pFliC	PBR322 harboring *fliC*	[6]
pMSD2	pJY26 harboring *espA*	[54]
pMSD3	pACYC184 harboring *espB*	[54]
pEspD	pCCCD3 harboring *espD*	This study
pCVD446	pJAY1512 harboring *escN*	[55]

## Data Availability

All data supporting the findings of this study are available within the article and previous publications. The investigators of this project are fully committed to sharing the specific reagents such as bacterial strains and mutants, and antisera with other scientists upon request by Material Transfer Agreement.

## Supplementary Materials

The following supporting information can be downloaded at: https://www.mdpi.com/article/10.3390/ijms232213705/s1.
