# Correction: Soria-Bustos et al. The Assembly of Flagella in Enteropathogenic *Escherichia coli* Requires the Presence of a Functional Type III Secretion System. *Int. J. Mol. Sci.* 2022, *23*, 13705

**DOI:** 10.3390/ijms27209105

**Published:** 2026-10-14

**Authors:** Jorge Soria-Bustos, Zeus Saldaña-Ahuactzi, Partha Samadder, Jorge A. Yañez-Santos, Ygnacio Martínez Laguna, María L. Cedillo-Ramírez, Jorge A. Girón

**Affiliations:** 1 Escuela de Biología, Benemérita Universidad Autónoma de Puebla, Puebla 72410, Mexico; jorge_s41@hotmail.com; 2 Department of Immunobiology, University of Arizona, Tucson, AZ 85721, USA; zeus_drago@hotmail.com (Z.S.-A.); partha@email.arizona.edu (P.S.); 3 Centro de Investigación en Ciencias Microbiológicas, Benemérita Universidad Autónoma de Puebla, Puebla 72410, Mexico; ignacio.martinez@correo.buap.mx; 4 Facultad de Estomatología, Benemérita Universidad Autónoma de Puebla, Puebla 72410, Mexico; j_antonio_yanez@yahoo.com; 5 Centro de Detección Biomolecular, Benemérita Universidad Autónoma de Puebla, Puebla 72410, Mexico; lcedil9@gmail.com

**Error in Figure 4**

In the original publication [1], there was a concern regarding the *rrsB* gel images presented in Figure 4A,B, which appeared similar. Because the original primary data used to generate this figure are no longer available, the authors are unable to verify the original image files or determine the reason for this apparent similarity. Therefore, in agreement with the Editorial Office and in the interest of maintaining transparency in the published record, Figure 4 and the corresponding text describing the expression analysis in Section 2.3 and the RT-PCR assay in Section 4.6 have been removed from the manuscript.

The authors state that this correction does not affect the scientific conclusions of the manuscript, as the data presented in Figure 4 were complementary to the main findings and were not essential to the overall conclusions of the study. This correction was approved by the Academic Editor. The original publication has also been updated.
