# Supplementary figures and images for: The Assembly of Flagella in Enteropathogenic Escherichia coli Requires the Presence of a Functional Type III Secretion System
Source: Int J Mol Sci. 2022 Nov 8;23(22):13705. doi: 10.3390/ijms232213705 (PMC9694695; doi:10.3390/ijms232213705)

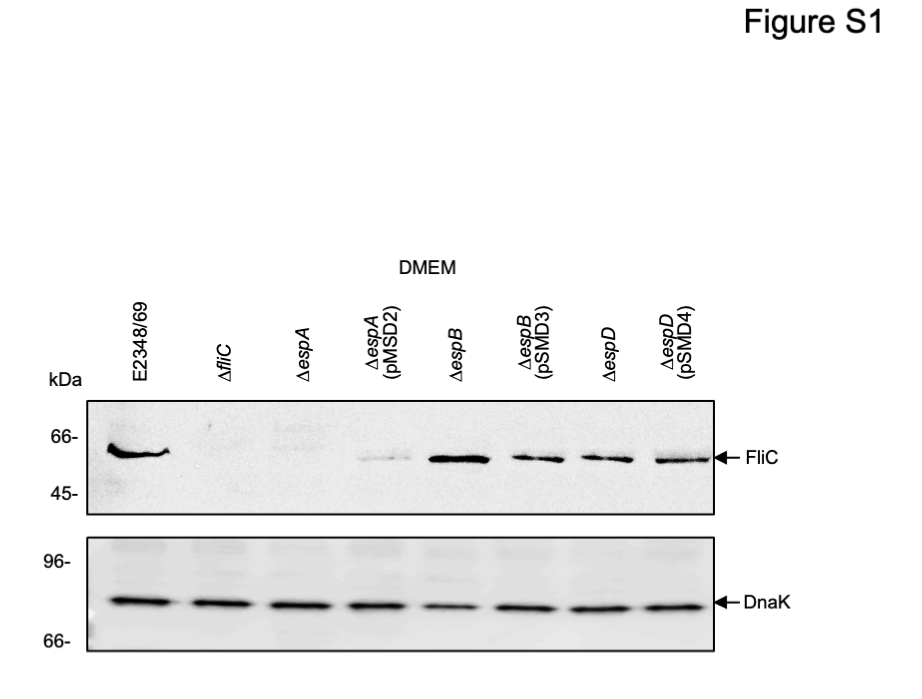

Supplement: Supplementary file 1 [file ijms-23-13705-s001.zip › ijms-2015301-Figure S1.tiff]
